# Cysteine coordination of Pb(II) is involved in the PbrR-dependent activation of the lead-resistance promoter, P*pbrA*, from *Cupriavidus metallidurans* CH34

**DOI:** 10.1186/1471-2180-12-109

**Published:** 2012-06-18

**Authors:** Jon L Hobman, Daniel J Julian, Nigel L Brown

**Affiliations:** 1 School of Biosciences, University of Birmingham, Edgbaston, Birmingham, B15 2TT, UK; 2 Present address: School of Biosciences, The University of Nottingham, Sutton Bonington Campus, Loughborough, LE12 5RD, UK; 3 Present address: Senior Vice-Principal's Office, The University of Edinburgh, Charles Stewart House, 9-16 Chambers Street, Edinburgh, EH1 1HT, UK

**Keywords:** Metal-resistance, Metal-protein interactions, Metalloregulation, Bacterial gene expression

## Abstract

**Background:**

The *pbr* resistance operon from *Cupriavidus metallidurans* CH34 plasmid pMOL30 confers resistance to Pb(II) salts, and is regulated by the Pb(II) responsive regulator PbrR, which is a MerR family activator. In other metal sensing MerR family regulators, such as MerR, CueR, and ZntR the cognate regulator binds to a promoter with an unusually long spacer between the −35 and −10 sequences, and activates transcription of resistance genes as a consequence of binding the appropriate metal. Cysteine residues in these regulators are essential for metal ion coordination and activation of expression from their cognate promoter. In this study we investigated the interaction of PbrR with the promoter for the structural *pbr* resistance genes, P*pbrA*, effects on transcriptional activation of altering the DNA sequence of P*pbrA*, and effects on Pb(II)-induced activation of P*pbrA* when cysteine residues in PbrR were mutated to serine.

**Results:**

Gel retardation and footprinting assays using purified PbrR show that it binds to, and protects from DNase I digestion, the P*pbrA* promoter, which has a 19 bp spacer between its −35 and −10 sites. Using β-galactosidase assays in *C. metallidurans*, we show that when P*pbrA* is changed to an 18 bp spacer, there is an increase in transcriptional activation both in the presence and absence of Pb(II) salts up to a maximum induction equivalent to that seen in the fully-induced wild-type promoter. Changes to the −10 sequence of P*pbrA* from TTAAAT to the consensus *E. coli −*10 sequence (TATAAT) increased transcriptional activation from P*pbrA*, whilst changing the −10 sequence to that of the Tn*501 mer* promoter (TAAGGT) also increased the transcriptional response, but only in the presence of Pb(II). Individual PbrR mutants C14S, C55S, C79S, C114S, C123S, C132S and C134S, and a double mutant C132S/C134S, were tested for Pb(II) response from P*pbrA*, using β-galactosidase assays in *C. metallidurans*. The PbrR C14S, C79S, C134S, and C132S/C134S mutants were defective in Pb(II)-induced activation of P*pbrA*.

**Conclusions:**

These data show that the metal-dependent activation of PbrR occurs by a similar mechanism to that of MerR, but that metal ion coordination is through cysteines which differ from those seen in other MerR family regulators, and that the DNA sequence of the −10 promoter affects expression levels of the lead resistance genes.

## Background

Lead (Pb) is a widely distributed, environmentally persistent, toxic metal. Most bacteria that are tolerant or resistant to lead either precipitate Pb in an insoluble form, or actively export it [1]. Although some metal efflux ATPases, such as ZntA from *Escherichia coli*, and CadA from *Staphylococcus aureus* plasmid pI258, can export Pb(II) as well as Zn(II) and Cd(II) [2,3], the only characterized bacterial Pb(II) specific resistance system is from *Cupriavidus* (formerly *Wautersia* and *Ralstonia*) *metallidurans* CH34 [4,5] - a Gram-negative, multiply metal-resistant, β-proteobacterium originally isolated from a decantation basin at a Belgian zinc production plant (and originally identified as *Alcaligenes eutrophus* CH34; [6]). Over 150 genes in CH34 are involved in metal resistance, of which at least 70 are carried on the plasmids pMOL28 (171 kb) or pMOL30 (234 kb), and the remainder are carried on the 3.92 Mb chromosome or on a 2.58 Mb second chromosome [7]. Plasmid pMOL30 carries the *czc* (Cd(II), Zn(II), Co(II)), *mer* (Hg(II)), *sil* (Ag(I)), *cop* (Cu(II)) and *pbr* Pb(II) resistance operons [4,8].

The *pbr* lead resistance operon from pMOL30 was originally predicted to contain structural genes which encode PbrT, a putative Pb(II) uptake protein belonging to the ILT (Iron Lead Transporter) family [9], a P-type efflux ATPase (PbrA), a predicted inner–membrane protein (PbrB), a predicted prelipoprotein signal peptidase PbrC and a Pb(II) binding protein, PbrD. The regulator of the *pbr* operon was shown to be PbrR, which is a MerR family regulator [4,10] Subsequent work has shown that the *pbr* operon also contains an interrupted orf; *pbrU* upstream of *pbrT*[11,12] which is predicted to encode a putative inner membrane (Major Facilitator Family MFS1) permease gene, which is probably inactive, but still part of the *pbr* operon; and that PbrB/PbrC is a fusion protein [11,12], and encodes an inner membrane bound undecaprenyl pyrophosphate (C_55_-PP) phosphatase [5]. The *pbr* operon contains a predicted MerR-like promoter from which *pbrRTU* are transcribed on one DNA strand, and the *pbrABCD* genes are transcribed as a polycistronic message on the other [4,12]. The most recent work on the mechanism of lead resistance encoded by the pMOL30 *pbr* operon has proposed a model where Pb^2+^ induces expression of the pMOL30-encoded PbrABCD by PbrR, as well as expression of zinc and cadmium efflux ATPase homologs ZntA and CadA which are carried on the chromosome or second chromosome. Each of these three ATPases is involved in exporting Pb^2+^ into the periplasm where inorganic phosphates produced by PbrB are involved in precipitating Pb^2+^ as insoluble lead phosphate. This model finds no role for PbrT, C, and D, yet some reports suggest PbrC may be required for the maturation or activity of phosphatase in the periplasm[5]. PbrR from pMOL30 (Rmet_5946) is related to several other PbrR-like regulators that have been identified in the *C. metallidurans* CH34 chromosome, including *pbrR2* (Rmet_2303 also known as *pbr691*[13,14] which is believed to regulate a *cadA* and a *pbrC* homolog on the chromosome, and *pbrR3* (Rmet_3456 also known as *pbr710*) believed to regulate a *zntA* homolog on the second chromosome, both of which are believed to be involved in Pb^2+^ export [12]. There is evidence for only very low levels of cross-regulation of the pMOL30 *PpbrA* promoter by PbrR2 or PbrR3 [15].

Other metal-sensing MerR family members include those responding to cadmium (CadR; [16,17]), copper (CueR; [18-20], ActP; [21], SctR; [22]), zinc (ZntR, [23,24]; ZccR (Zn, Co, Cd), [25]) and gold (GolS, [26]). Metal-sensing MerR family regulators share many common features: they bind to and activate gene expression from promoters with unusually long spacer sequences of 19-20 bp between the −35 and −10 sequences, and contain cysteine and other amino acids that are essential in coordinating metals and activating gene expression [10,16,20,27-29].

The objectives of this study were to 1) Characterize the interaction between PbrR and the *pbrA* promoter, and study the effects on transcription of shortening the 19 bp spacer between the −35 and −10 sequences, and altering the −10 sequence of *PpbrA*; and 2) to investigate the importance of cysteine residues in PbrR activation of P*pbrA* in response to Pb(II) ions. To this end each of the cysteine residues in PbrR (C14, C55, C79, C114, C123, C132 and C134) were individually changed to serine residues and a double mutant (C132S, C134S) was created. The effects of these mutations on *in vivo* transcriptional activation in response to Pb(II) were determined in *C. metallidurans* using β-galactosidase assays.

## Methods

### Bacterial strains, plasmids and growth media

Bacterial strains and plasmids used in this study are shown in Table 1. *Escherichia coli* strains were grown in LB broth [30] at 37°C. *C. metallidurans* strains were grown at 30°C in 869 medium, 284 Tris or 284 MOPS medium [4,6]. For β-galactosidase assays of PbrR-regulated P*pbrA* promoter activity, *C. metallidurans* strains were grown in 284 MOPS medium [4] minimising any Pb(II) precipitation during growth. *C. metallidurans* strains were grown in SOB medium without MgSO_4_[30] prior to electroporation of plasmids, and SOB medium containing MgSO_4_ after electroporation. Pb(II) induction was achieved by growth in PbNO_3_, and antibiotics were used at the following concentrations:- for *E. coli*: carbenicillin (Melford laboratories, UK), 200 μg/ml; chloramphenicol 25 μg/ml; kanamycin, 50 μg/ml and trimethoprim lactate 30 μg/ml (all from Sigma Chemical UK); for *C. metallidurans*: trimethoprim lactate 500 μg/ml.

**Table 1:** Bacterial strains and plasmids

**Bacterial strain**	**Properties or Genotype**	**Reference or source**
*E. coli*		
TG2	*sup*E *hsd*Δ5 thiΔ (*lac-pro*AB) *F'* Δ(*srl-rec*A)306::Tn*10*(Tc^r^) *lacZ*Δ*M15*	[31]
BL21(DE3)pLysS	F^-^*ompT hsdS*_*B*_ (r_B_- m_B_-) *gal dcm* (DE3) pLysS (Cm^r^)	Novagen
*C. metallidurans*		
CH34	Zn, Cd, Co, Pb, Cu, Hg, Ni and Cr resistance	[6]
AE104	Plasmid-cured *C. metallidurans* strain- sensitive to toxic metals	[6]
Plasmid	Description	Reference or source
pET32LIC	Ap^r^ Overexpression plasmid for ligation-independent cloning	Novagen
pET32LIC *pbrR*	Ap^r^*pbrR* cloned into pET32LIC	This study
pMa5/8	Ap^r^ Cm^s^ Mutagenesis vector	[32]
pMc5/8	Ap^s^ Cm^r^ Mutagenesis vector	[32]
pMaPbrR/P*pbrA*	Ap^r^ Cm^s^ Mutagenesis vector with *pbrR/PpbrA* cloned in to it	This study
pMOL1139	Km^r^, The *pbr* operon cloned into plasmid pRK415	B. Borremans
pMU2385	Tp^r^ 13.3 kb low copy number *lacZ* reporter plasmid	[33]
pMUP*pbrA*	Tp^r^ pMU2385 containing the PpbrA promoter directing *lacZ* transcription	This study
pMUP*pbrA*-1	Tp^r^ pMU2385 containing the PpbrA promoter with a 1 bp deletion	This study
pMUP*pbrA*con	Tp^r^ As pMU*PpbrA*, but −10 sequence changed to *E. coli* consensus	This study
pMUP*pbrA*mer	Tp^r^ As pMU*PpbrA*, but −10 sequence changed to *mer* promoter	This study
pMUPbrR/P*pbrA*	Tp^r^, pMU2385 containing *pbrR*, *PpbrA ΔpbrA* directing *lacZ* transcription	This study
pMUPbrRC14S/P*pbrA*	As pMUPbrRP*pbrA*, but PbrR C14S	This study
pMUPbrRC55S/P*pbrA*	As pMUPbrRP*pbrA*, but PbrR C55S	This study
pMUPbrRC79S/P*pbrA*	As pMUPbrRP*pbrA*, but PbrR C79S	This study
pMUPbrRC114S/P*pbrA*	As pMUPbrRP*pbrA*, but PbrR C114S	This study
pMUPbrRC132S/P*pbrA*	As pMUPbrRP*pbrA*, but PbrR C132S	This study
pMUPbrRC134S/P*pbrA*	As pMUPbrRP*pbrA*, but PbrR C134S	This study
pMUPbrRC132,134 S/P*pbrA*	As pMUPbrRP*pbrA*, but PbrR C132S/C134S	This study
pUC21	Ap^r^, high copy number cloning vector; ColE1 replicon	[34]
pUK21	Km^r^, intermediate copy number cloning vector; p15A replicon	[34]
pUK21*pbr*1	Km^r^, *Hind*III/*Sal*I *pbrR*/P*pbrA*/Δ*pbrA* from pMOL1139 cloned into pUK21	This study

### DNA manipulations

DNA manipulations were as described by [30]. Oligonucleotides were synthesized by Alta Bioscience, the University of Birmingham; or MWG Biotech, Germany. The DNA sequence of all mutants and cloned PCR products were confirmed by sequencing using a PE Applied Biosystems Big Dye version 2.0 sequencing kit according to the manufacturer’s protocol, followed by analysis on an ABI 3700 sequencer in the Functional Genomics Laboratory, School of Biosciences, the University of Birmingham. The primers used for sequencing were: pMUforward and pMUreverse, complementary to the sequences flanking the multiple cloning site of pMU2385, and *PbrA*pe for pMa*pbrR*/P*pbrA* clones (Table 2).

**Table 2:** Oligonucleotides used for site directed mutagenesis, and overexpression

**Oligonucleotide**	**Sequence**	**Description or reference**
*pbrR C14S*	5’ CCA CCG GGG ATG CGG TGC 3’	PbrR mutagenesis primer
*pbrR* C55S	5’ CCA GAG ACC GGG AGT GAC G 3’	PbrR mutagenesis primer
*pbrR* C79S	5’ GAC TTC ACC GGA ATC CTG G 3’	PbrR mutagenesis primer
*pbrR* C114S	5’ GGC ACC AGA AGA GGC TTC G 3’	PbrR mutagenesis primer
*pbrR* C123S	5’ GCA GAA TCC CGG ACG ATT G 3’	PbrR mutagenesis primer
*pbrR* C132S	5’ CGT ATC ACA CAC GGA GTC CGA C 3’	PbrR mutagenesis primer
*pbrR* C134S	5’ CGT ATC AGA CAC GCA GTC CGA C 3’	PbrR mutagenesis primer
*pbrR* C132S/C134S	5’ CGT ATC AGA CAC GGA GTC CGA C 3’	PbrR mutagenesis primer
*pbrA*pe	5’ GCG CCA ACC GTG CTC GGT TCT GGG 3’	Primer extension/sequencing primer [4]
pbrBstEII	5’ GCG AAT GGT CAC CAC CGG 3’	Primer to amplify *PpbrA*
pbrNruI	5’ GCT TGT CGC GAA TCA GCG 3’	Primer to amplify *PpbrA*
pMU forward	5’ GAT TCT CCC CAC ATC ACC AG 3’	Sequencing primer for pMU2385
pMU reverse	5’ TGC CAG CAT TTC ATA ACC AA 3’	Sequencing primer for pMU2385
M13-F	5’ CGC CAG GGT TTT CCC AGT CAC GAC 3’	Sequencing primer for pUK plasmids
M13-R	5’ GAG CGG ATA ACA ATT TCA CAC AGG 3’	Sequencing primer for pUK plasmids
con*pbr*:	5’ CTAGAGGGTTAATCGGCAAC 3’	P*pbrA* mutagenesis primer
mer*pbr*:	5’ CTAGAGGGTGTAAGGTCGGCAAC 3’	P*pbrA* mutagenesis primer
-1EcoPbr	5’ GGG GAA TTC GAA GCT TGC T 3’ (3’ primer)	P*pbrA* mutagenesis primer
-1CentreBam	5’ GCC GAT TTA AAC CCT CTA GT 3’ (primer B)	P*pbrA* mutagenesis primer
-1CentreEco	5’ CGG CTA AAT TTG GGA GAT CA 3’ (primer A)	P*pbrA* mutagenesis primer
-1BamPbr	5’ CAG TAT ACC TAG GCA GCT GG 3’ (5’ primer)	P*pbrA* mutagenesis primer
*pbrR* ATG (LIC)	5’ GAC GAC GAC AAG ATG AAT ATC CAG ATC GGC GAG C 3’	PbrR cloning and overexpression primer
*pbrR* TAG (LIC)	5’ GAG GAG AAG CCC GGT CTA GTC GCT TGG ATG GGC 3’	PbrR cloning and overexpression primer
T7 terminator	5’ CGA TCA ATA ACG AGT CGC C 3’	Sequencing primer

Underlined bases highlight alteration from the wild-type sequence.

### PbrR overexpression and purification

The *pbrR* gene was amplified from pMOL1139 using Vent_R_® DNA polymerase (New England Biolabs) and the primers: pbrRATG (LIC) and pbrRTAG (LIC) (Table 2). The *pbrR* PCR product was annealed with plasmid pET32LIC (Novagen), according to manufacturers’ recommendations. DNA sequencing using the primer T7 reverse (Table 2) was used to confirm the nucleotide sequence of the cloned fragment. The thioredoxin-PbrR fusion protein was overexpressed in *E. coli* BL21 (DE3) pLysS, purified and stored under reducing conditions as described in [23]. The thioredoxin- S tag was cleaved from the fusion protein using enterokinase, according to the manufacturer’s protocol (Novagen) and removed using S-tag affinity agarose. PbrR purity was estimated by PAGE analysis. The concentration of the purified protein was determined by Bradford assay [35].

### Gel retardation and DNAse I protection assays of P*pbrA* with PbrR

**Figure 1:**
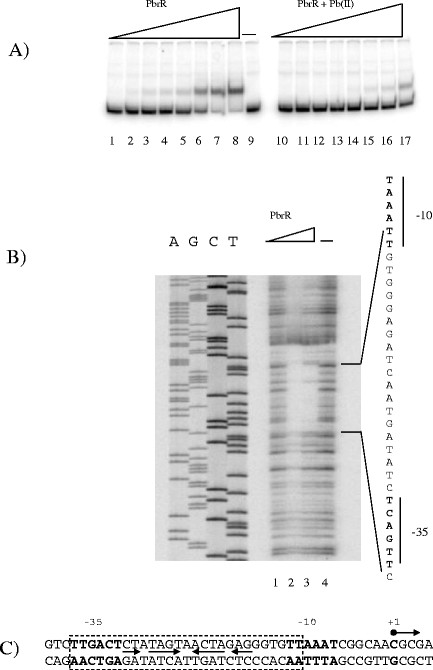
**(a) Gel retardation of P*pbrA*with PbrR.** Each reaction contained the same amount of ^32^P-end-labelled 296 bp P*pbrA* PCR product (60 fmol). Lanes 1, 9 and 10 contained no PbrR. PbrR concentrations in lanes 2–8 and 11–17 increase 2-fold from 0.3 to 19.2 pmol. Lanes 10–17 contained 10 μM Pb(II). (**b**) DNase I protection assay of PbrR bound to the 296 bp PCR product containing the PbrA promoter. Lanes AGCT, DNA sequence of the 296 bp PCR product pbrA promoter, using the pbrApe primer. Lanes 1 and 4, no added pbrR, lane 2 and 3 increasing amounts of added PbrR. (**c**) Diagram of the P*pbrA* promoter. The transcript start site is marked in bold and indicated with an arrow [4]. The region of the promoter protected by PbrR from DNAase I digestion is marked with a box. The predicted −35 and −10 sequences are marked in bold, and the dyad symmetrical sequence is marked with arrows.

Gel retardation experiments were as described in [36], with initial experiments to determine PbrR DNA binding using a 1144 bp *Hin*dIII/*Sal*I fragment from pMOL1139 containing *pbrR*, P*pbrA* and a truncated *pbrA* (positions 409 and 1553 on the *pbr* operon) [4] cloned into pUK21 [34] to make plasmid pUKpbr1. pUKpbr1 was digested with *Nru*I/*Bst*EII and end labelled with [γ^32^P]-dATP for gel retardations. Further gel retardation and footprinting experiments used a 296 bp P*pbrA* PCR product, amplified from pMOL1139 using the primers pbr*Bst*EII and pbr*Nru*I (Table 2) and labelled using [γ^32^P]-dATP. DNAse I protection assays of P*pbrA* with PbrR were as described by [37], using the 296 bp P*pbrA* promoter PCR product detailed above. The DNA sequence of the region was obtained from the 296 bp P*pbrA* PCR product using the *pbrA*pe primer (Table 2) [4] and run alongside the DNAase I footprint (Figure 1B).

### Cloning of *pbrR*-P*pbrA*-Δ*pbrA* and mutagenesis of the PbrR cysteines

All cloning and mutagenesis work was done in *E. coli* K-12 TG2. The 1144 bp *pbrR-*P*pbrA-*Δ*pbrA* DNA fragment described above was cloned into pMa5/8 [32] from pUK21pbr1 using the flanking *Eco*RI and *Bam*HI sites to make pMaPbrR/P*pbrA*. Gapped duplex mutagenesis of each of the cysteine residues in pbrR was as previously described [32] using the primers *pbrR*C14S, *pbrR*C55S, *pbrR*C79S, *pbrR*C114S, *pbrR*C123S, *pbrR*C132S, *pbrR*C134S, or *pbrR*C132S, C134S (Table 2), and mutants verified by DNA sequencing as described [15]. The wild type and mutant *pbrR* genes on the 1144 bp *pbrR-*P*pbrA-*Δ*pbrA* DNA fragment were individually sub-cloned as *Eco*RI - *Bam*HI fragments into pMU2385 [33] as described previously [15]. The resulting constructs contained a self-regulating transcriptional unit, with PbrR controlling the transcription of *pbrR* through P*pbrR* and regulating transcription of *lac*Z in pMU2385 on the other DNA strand through P*pbrA*. These constructs were the basis of the studies of the regulation of P*pbrA* by PbrR in *C. metallidurans* AE104.

### Cloning and mutagenesis of P*pbrA*

A 266 bp *Sph*I - *Nru*I fragment containing the P*pbrA* promoter (positions 1062 and 1328 of the *pbr* operon) was cloned from pMOL1139, into the *Hin*dIII site of pUK21, by rendering the vector and insert blunt-ended using T4 DNA polymerase. The cloned P*pbrA* DNA fragment was sub-cloned as an *Eco*RI - *Bam*HI fragment into pMa5/8 for site directed mutagenesis. The −10 sequence of P*pbrA* was mutated as described above using the primers con*pbr* and mer*pbr* (Table 2) to change the P*pbrA −*10 sequence from TTAAAT (wild type) to TATAAT (consensus) or TAAGGT (*mer*-like). The mutant P*pbrA* promoters were cloned into pMU2385 using *Eco*RI and *Bam*HI, creating plasmids pMUP*pbrA*, pMUP*pbrA*(con) and pMUP*pbrA*(mer) in which the *pbrA* promoter regulates expression of the *lacZ* gene. After DNA sequencing, the activity of these mutant promoters was assayed in *C. metallidurans* CH34.

### Construction of the P*pbrA −*1 mutant

Mutagenic PCR [38] of the 1144 bp *pbrR-*P*pbrA-ΔpbrA* DNA fragment from pMa*pbrR*/P*pbrA* was used to construct the −1 promoter mutant of P*pbrA*, using the primers -1CentreEco and -1CenterBam to introduce the −1 deletion, and primers -1EcoPbr and -1BamPbr as flanking primers (Table 2). The PCR product containing the -1P*pbrA* promoter was digested with *Eco*RI and *Bam*HI and subcloned into the multiple cloning site of pMU2385. The DNA sequence of the *pbrR-*P*pbrA-*Δ*pbrA* DNA fragment containing the −1 deletion in *PpbrA* was confirmed, and this plasmid provided the mutant promoter for the assay in *C. metallidurans* AE104.

### β-galactosidase assays in *C. Metallidurans*

pMU2385 plasmid constructs were electroporated into *C. metallidurans*, and cultures containing pMU2385 derivatives were assayed for ß-galactosidase activity as described in [39] with modifications described by [15].

## Results

### PbrR binds to the *pbrA* promoter and pb(II) decreases the binding affinity of PbrR to P*pbrA* in vitro

PbrR was overexpressed as a thioredoxin-his Tag-S tag-fusion protein using the pET32-LIC expression system, purified and released after enterokinase digestion as untagged, full length PbrR, as described in Materials and Methods. The PbrR preparation was estimated as being >95% pure PbrR by Coomassie Blue staining of standard SDS-PAGE gels (data not shown). We had originally identified a candidate P*pbrA* promoter based on sequence similarity to other MerR family promters, and on run-off transcription studies of the *pbr* operon [4] and studied PbrR interactions with this region of the *pbr* operon. Initial PbrR gel retardation assays on ^32^P-end-labelled DNA from pUK21pbr1, which contained *pbrR*/P*pbrA*/Δ*pbrA*, had been digested with *Bst*EII and *Nru*I showed retardation only of the 282 bp *Bst*EII/*Nru*I DNA fragment containing the previously identified P*pbrA* promoter region, and no other fragments from the plasmid (data not shown). Addition of PbrR to the end-labelled 296 bp P*pbrA* PCR product retarded this fragment, and addition of Pb(II) to PbrR and P*pbrA* increased the amount of PbrR required to retard the P*pbrA* DNA fragment (Figure 1A) indicating that PbrR-Pb(II) had a lower affinity *in vitro* with P*pbrA* than did apo-PbrR did, as is the case with MerR and Hg(II) (reviewed in [10]).

### PbrR protects the *pbrA* promoter from DNAse I digestion *in vitro*

The 296 bp P*pbrA* PCR product described above was also used to determine the PbrR binding site on the promoter by DNase I protection assay. Figure 1B shows the autoradiograph of the PbrR DNase I footprint on P*pbrA*. The region protected by PbrR on P*pbrA* includes the −35 and −10 sequences as well as the 19 bp spacer containing an imperfect dyad symmetrical sequence between them, and is consistent with DNAse I protection results for MerR, CueR and ZntR [18,20,23,24,40].

**Figure 2:**
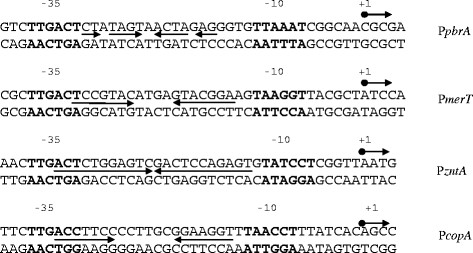
**Alignment of selected promoters for structural genes regulated by MerR family metal responsive regulators: PbrR**[4]**; MerR**[10]**, ZntR**[23]**, CueR**[20]. The −35 and −10 sequences are marked in **BOLD**. Arrows show dyad symmetrical DNA sequences within the promoters.

The transcription start site [4], the predicted −35 and −10 sites, and the region of the P*pbrA* promoter protected by PbrR are shown in Figure 1C. The P*pbrA* promoter has a −35 sequence (TTGACT) that is identical to those for P*merT* from Tn*501* and P*zntA* from *E. coli* K-12 (Figure 2) and shares 5/6 identity with the consensus *E. coli −*35 sequence. The predicted P*pbrA −*10 sequence (TTAAAT) has a 4/6 identity to the consensus *E. coli −*10 sequence (TATAAT) and the spacing between the −35 and −10 sequences is 19 bp, as is the case with other MerR family regulatory regions except ZntR (20 bp; [23]).

### Promoter DNA mutations alter P*pbrA* activity in *C. Metallidurans*

**Figure 3:**
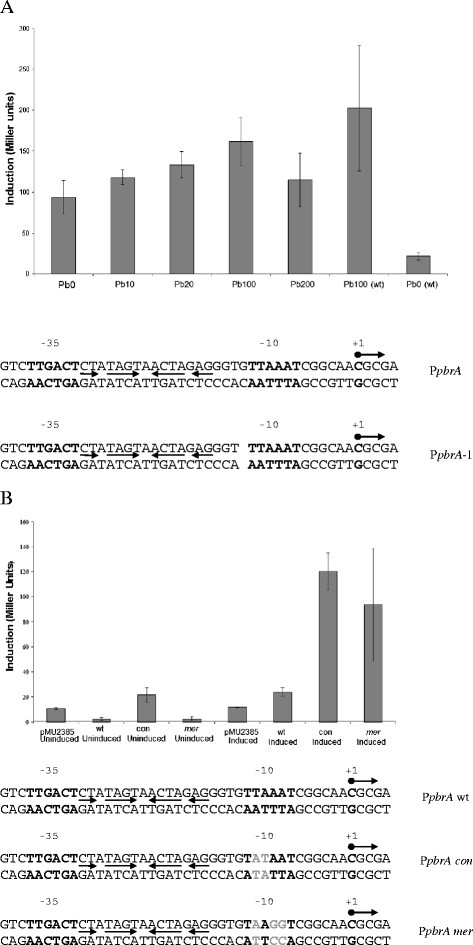
**(A) β-galactosidase assay measurement of the activation of P*pbrA*, containing a 1 nt deletion in the 19 bp promoter spacer, to increasing levels of Pb(II) in*C. metallidurans*AE104 carrying pMUPbrR*pbrA*-1.** Micromolar Pb(II) concentrations are indicated by the suffix to Pb on the abscissa. Pb0 contains no added Pb(II), Pb200 contains 200 μM Pb(II) . The sequence of wild-type P*pbrA* and the −1 mutant P*pbrA* are shown below the graph. The −35 and −10 sequences are marked in **BOLD**. Arrows show dyad symmetrical DNA sequences within the promoters. (**B**) β-galactosidase assay measurement of the activation of −10 sequence mutant P*pbrA* clones in pMU2385 in response to no added Pb(II) or 100 μM Pb(II). WT denotes wild-type −10 sequence (TTAAAT), CON denotes the *E. coli* consensus promoter −10 sequence (TATAAT) and MER the Tn*501* P*merT* promoter −10 sequence (TAAGGT). The sequences of the wild-type (P*pbrA* wt), consensus (P*pbrA con*), and P*merT*-like promoters (P*pbrA mer*) are shown below the graph. The −35 and −10 sequences are marked in **BOLD**. Arrows show dyad symmetrical DNA sequences within the promoters, and altered bases are marked in Gray.

The importance to promoter functionality of the number of nucleotides between the −35 and −10 sequences of the P*pbrA* promoter, and the effects of altering the DNA sequence of the PbrR binding site or −10 sequence of P*pbrA* were investigated using pMUPbrR/*PpbrA −*1 in *C. metallidurans* AE104. The P*pbrA −*1 mutant (Figure 3A), in which the spacer between the −35 and −10 sequences was shortened in such a way that the −35 and −10 sequences were not altered, and the dyad symmetrical sequences in the spacer between the −35 and −10 were retained, showed increased promoter activity in the absence of Pb(II) (Figure 3A) compared to the wild type promoter, but no induction beyond the maximum level seen for the wt promoter with 100 μM Pb(II). These results are similar to those seen for the MerR activated promoter P*merT −*1 from Tn*501*[41], which is constitutively transcriptionally active in both the presence and absence of Hg(II). Changes to the *pbrA* promoter −10 sequence, so that it more closely resembled the consensus sequence for an *E. coli* promoter [42], caused up-regulation of P*pbrA* activity both in the absence and presence of Pb(II). Changes made in P*pbrA* so that it resembled the Tn*501 merT* promoter −10 sequence resulted in promoter activity remaining repressed in the absence of Pb(II), but strongly induced in its presence to expression levels 5-fold higher than the wild-type *pbrA* promoter (Figure 3B). These differences in promoter sequence are likely to alter RNA polymerase binding to the promoter, which could in turn affect the structure of the PbrR-RNA polymerase-DNA ternary complex.

### Cysteines 14, 79 and 134 in PbrR are essential for pb(II) responsive transcription from P*pbrA* in *C. Metallidurans* AE104

**Figure 4:**
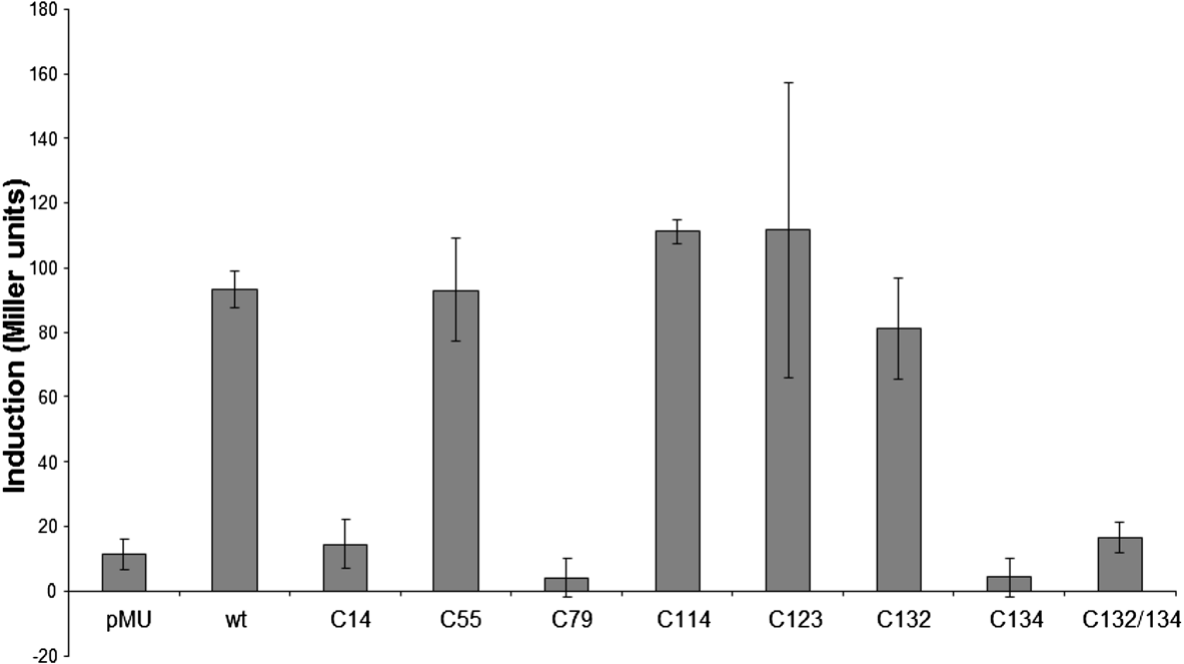
**β-galactosidase assays in*C. metallidurans*AE104 of P*pbrA*activation in response to 20 μM Pb(II) on wild-type PbrR and its cysteine mutants in pMUPbrR/P*pbrA.***

pMUPbrR/P*pbrA* derivatives carrying PbrR cysteine mutants (C14S, C55S, C79S, C114S, C123S, C132S, C134S, and C132S/C134S) (Table 1) were assayed for Pb(II) –dependent induction of the *pbrA* promoter in *C. metallidurans* AE104, which did not carry pMOL28 or pMOL30. These were grown in a sublethal concentration of Pb(II) (20 μM) which was sufficient to activate expression from P*pbrA*, without affecting growth of the Pb(II) sensitive AE104 strain. β-galactosidase assays of wild type and cysteine mutant PbrR responses to Pb(II) in *C. metallidurans* AE104 (Figure 4) showed cysteines C14, C79, and C134 were essential for Pb(II) induced transcriptional activation of P*pbrA* by PbrR. The double mutant C132S, C134S also lost Pb(II) induced activation of transcription from P*pbrA*, consistent with the result for the single C134S mutant.

## Discussion

PbrR is a member of the MerR family of regulators which sense metals and other environmental stimuli, and activate gene expression in response to these signals. The archetype of the family, MerR, regulates both its own expression and expression of the mercuric ion resistance genes in the polycistronic *mer* operon from a divergent promoter: P*mer*. MerR activates expression of the structural genes at the P*merT* operator/promoter (*o/p*) site, which has an unusually long spacer of 19 bp between the −35 and −10 sequences of the promoter (compared to the consensus *E. coli* σ^70^ promoter spacing of 16-18 bp [10]). The MerR dimer binds to a dyad-symmetrical DNA sequence within the spacer, and when three essential cysteine residues (C89, C117 and C126) in the MerR dimer coordinate to a mercuric ion in a trigonal coordination [28,29] bridging between each MerR homodimer, this change in MerR homodimer interaction is transmitted to the promoter, causing an allosteric underwinding of ~33^O^ of the DNA at the *o/p* site, which realigns the −35 and −10 sequences of the promoter so that σ^70^ RNA polymerase can contact the promoter sequences forming the transcription open complex [43,44].

PbrR from *C. metallidurans* CH34 plasmid pMOL30 binds to and protects from DNAase I digestion the predicted P*pbrA* operator/promoter (Figure 1) (4). P*pbrA* has striking similarities to other metal ion-responsive MerR family promoters (Figure 2). Assays of P*pbrA* mutants where the spacing between the −10 and −35 sites are shortened to 18 bp, whilst the internal dyad symmetry is maintained, showed that PbrR-induced expression from *PpbrA* is upregulated even in the absence of Pb(II) (Figure 3). These data are all consistent with the model of activation for the MerR promoter [41,43,44]. Change of the DNA sequence of the −10 element of P*pbrA* to either the consensus *E. coli* promoter −10 sequence or the Tn*501* P*merT* promoter −10 sequence also caused up-regulation of promoter activity, although the P*pbrA*/Tn*501 PmerT*-like promoter still retained Pb(II) repression and induction, rather than a constitutive up-regulation seen in the −10 consensus promoter mutant. These data emphasize the importance of individual nucleotides within the promoter in affecting promoter strength, and indicate that P*pbrA* is suboptimal for maximum induction of the structural *pbr* genes. It is possible that this may represent a mechanism for fine-tuning of expression of the *pbr* structural genes.

**Figure 5:**
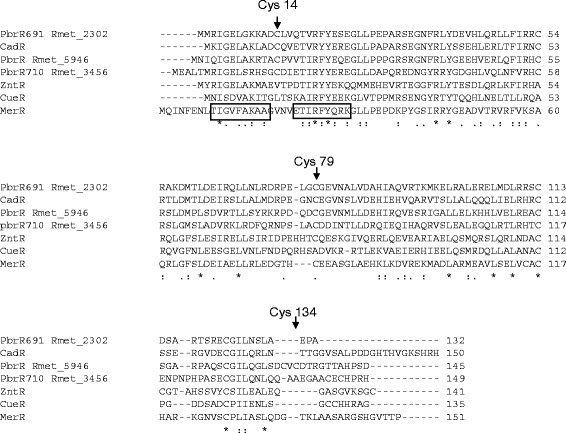
**ClustalW**[47,48]**alignment of metal sensing MerR regulators.** PbrR (Rmet_5946), PbrR691 (Rmet_2302) and PbrR710 (Rmet_3456) are from the genome of *C. metallidurans* CH34. CadR is from *Pseudomonas stutzeri* A1501. ZntR, and CueR are from the *E. coli* K-12 genome, and MerR is from Tn*501*. The helices of the Helix-Turn-Helix DNA binding domain are boxed. Essential cysteine residues (Cys14, Cys79, and Cys134 –PbrR numbering) required for activation of P*pbrA* by PbrR are marked. Key to symbols: * = residues in that column are identical in all sequences in the alignment. The symbol : = conserved substitutions have been observed, and the symbol . = semi-conserved substitutions are observed.

In other metal ion-sensing MerR family regulators, cysteine residues are essential for metal coordination and functionality. *In vivo* assays of the activity of cysteine to serine mutant PbrR proteins in *C. metallidurans* AE104 (which lacks pMOL30) have shown that C14, C79 and C134 are essential for PbrR Pb(II) sensing and activation of P*pbrA* (Figure 4). PbrR C14 lies in the turn of the predicted helix-turn-helix DNA binding domain of PbrR (Figure 5) and a change of amino acid at this point could disrupt the binding of PbrR to P*pbrA*. Mutants in the second helix of this region of MerR have lost both activation and repression activity [45,46]. The loss of Pb(II) response in the PbrR C79S mutant is consistent with the prediction from a structure-based sequence alignment that this residue is essential for discriminating between +1 and +2 charge ions, with a cysteine being found at this position in regulators that respond to +2 ions [27]. Mutagenesis studies have all identified a cysteine residue at this position as being essential for *in vivo* metal-dependant activation of expression in MerR, ZntR, and ZccR.

C134 in PbrR (Rmet_5496) is also essential for Pb(II) response and is part of a CVC (CXC) motif which is often found in PbrR regulators associated with orthologs of PbrABC, but not in the PbrR homologues PbrR2 (PbrR691 Rmet_2302) and PbrR3 (PbrR710 Rmet_3456), or CadR (Figure 5). A CVC motif is also found in the CadC repressor: alterations of either cysteine in this motif in CadC reduced or abolished sensing of Pb(II), Cd(II) and Zn(II) [49] and both cysteines are required for metal coordination [50,51]. Although C79 and C134 of the PbrR homodimer are essential for Pb(II) induction of P*pbrA*, the C132S mutant shows only a slightly reduced, not abolished, response to Pb(II). Pb(II) has been shown to have a preference for binding to cysteine residues in a tri-coordinate Pb(II)-thiol conformation [52], and Chen and coworkers have reported that the PbrR-related PbrR691 (PbrR2, Rmet_2302) regulator from the *C. metallidurans* genomic island 1 coordinates Pb(II) via 3 (possibly 4) cysteine coordination [14]. Pb(II) has been shown to coordinate in biological systems via a distorted trigonal planar geometry involving S and N coordination in a biomimetic N2S (alkylthiolate) compound [53], and the Pb(II), Cd(II) and Zn(II) response of the *S. aureus* pI258 cadmium resistance repressor CadC is dependent on three cysteine residues [49,54]. DNA footprinting suggests that like MerR, PbrR functions as a homodimer. It is possible that Pb(II) may coordinate to cysteine and histidine (or other N- side chain amino acid) residues or O-containing side chain amino-acid residues in the PbrR homodimer and C79 could provide the ligand for metal bridging between the homodimers, and in current models is thought to be necessary to trigger DNA underwinding at the regulated promoter [27]. There are histidine, glutamine, lysine and arginine residues in PbrR close to the metal-binding domain (Figure 5). In ZntR, each homodimer coordinates two zinc atoms per metal binding domain (MBD), one via C114 and C124 of the MBD, and C79 from the other monomer, whilst the other zinc atom is coordinated to C115 and H119 of the MBD, and C79 from the other monomer and both zinc atoms also coordinate to oxygen from a bridging phosphate [27,54]. Structural studies are required to understand further how Pb(II) coordinates to PbrR.

We cannot exclude the possibility that the PbrR C79S and C134S mutants we have made may have altered DNA-binding features, which may account for loss of Pb(II) response. However, mutants in the MBD of other MerR family regulators do not, but mutants in the helix-turn helix domain of these regulators do [45,46].

## Conclusion

The metal-responsive MerR family transcription activators can be classified into groups which sense Hg, or Cu/Ag/Au, or Zn/Cd/Pb, and several other phylogenetically-related but uncharacterized regulator clusters [55]. PbrR (Rmet_5946) and the related PbrR691 (R_met 2302) are unusual amongst the phylogenetic cluster of related Zn(II)/Cd(II)/Pb(II)-sensing MerR family regulators that have been tested for metal specificity, because they exclusively respond to Pb(II) in plasmid based assays in *C. metallidurans* (PbrR: [15,56]) or using FRET (PbrR691, [13]) without any transcriptional response to Zn or Cd, whereas related MerR family regulators that have been tested respond to a greater or lesser extent to Zn(II), Cd(II) and Pb(II) [10,23,57], as do SmtB/ArsR family repressors [47,54]. However, transcriptomics experiments indicate that the *pbr* structural genes are also induced in the presence of other metals, arguing that expression of the *pbr* operon and other metal resistance operons in *C. metallidurans* is influenced by other factors [7,12].

Our experiments show that the mechanism of transcriptional activation by PbrR appears to be essentially identical to that of MerR family regulators that have been characterized. PbrR contains three cysteine residues that are necessary for Pb(II)-induced transcription from the *pbrA* promoter. C14 is in the helix-turn-helix DNA binding domain, and may be essential for the regulator/DNA interaction. C79 is essential in all divalent metal ion responsive MerR regulators tested so far, whilst C134 is not found in other characterized MerR regulators. Our data show that PbrR transcription is activated by Pb(II) using different amino acids to other divalent metal ion-activated MerR regulators, but further work is required to determine whether Pb(II) coordinates other residues in PbrR.

## Abbreviations

Tp, Trimethoprim; Ap, Ampicillin; Km, Kanamycin.

## Competing interests

The authors declare that they have no competing interests.

## Authors’ contributions

JLH and DJJ carried out the experimental studies. JLH drafted the manuscript. NLB conceived and coordinated the study. All authors read and approved the manuscript.
